# Association between left ventricular longitudinal function and left atrial strain in left ventricular dysfunction

**DOI:** 10.1093/eschf/xvag046

**Published:** 2026-02-10

**Authors:** Björn Östenson, Elsa Bergström, Katarina Steding-Ehrenborg, Ashwin Venkateshvaran, Marcus Carlsson, Håkan Arheden, Ellen Ostenfeld

**Affiliations:** Clinical Physiology, Department of Clinical Sciences Lund, Lund University, Skåne University Hospital, Entrégatan 7, 221 85, Lund, Sweden; Clinical Physiology, Department of Clinical Sciences Lund, Lund University, Skåne University Hospital, Entrégatan 7, 221 85, Lund, Sweden; Clinical Physiology, Department of Clinical Sciences Lund, Lund University, Skåne University Hospital, Entrégatan 7, 221 85, Lund, Sweden; Clinical Physiology, Department of Clinical Sciences Lund, Lund University, Skåne University Hospital, Entrégatan 7, 221 85, Lund, Sweden; Clinical Physiology, Department of Molecular Medicine and Surgery, Karolinska Institutet, Karolinska University Hospital, Stockholm, Sweden; Clinical Physiology, Department of Clinical Sciences Lund, Lund University, Skåne University Hospital, Entrégatan 7, 221 85, Lund, Sweden; Clinical Physiology, Department of Clinical Sciences Lund, Lund University, Skåne University Hospital, Entrégatan 7, 221 85, Lund, Sweden

**Keywords:** Global longitudinal strain, CMR, Atrioventricular coupling, Ventricular function, Atrial function

## Abstract

**Introduction:**

Left ventricular (LV) longitudinal function is a prognostic marker of hospitalization and mortality in LV dysfunction. Recently, left atrial (LA) reservoir and conduit strain have also been presented as independent prognostic markers. However, the atria and ventricles are coupled in the fibrous atrioventricular plane (LA–LV coupling). The degree to which the LA strain is affected, or even determined, by the LV longitudinal function in LV dysfunction has been explored by echocardiography, but not by cardiac magnetic resonance imaging (CMR). Therefore, we aimed to quantify the association between LV longitudinal ventricular function and LA strain using CMR feature-tracking.

**Methods:**

Three hundred and forty-two patients with LV dysfunction (including heart failure with reduced ejection fraction (HFrEF), candidates for cardiac resynchronization therapy (CRT) implantation, and ischaemic heart disease (IHD)), and 19 healthy controls (HC) who had undergone CMR were retrospectively included. LV global longitudinal strain (LV-GLS), LV atrioventricular plane displacement (AVPD), and LA-GLS (i.e. reservoir strain) were analysed in long-axis views using CMR feature-tracking.

**Results:**

LA-GLS was lower in the LV dysfunction group when compared to HC (12 ± 8% vs 19 ± 7, *P* < .001), mirroring reductions in LV-GLS (−10 ± 5% vs −19 ± 3, *P* < .001), and LV-AVPD (9 ± 3 vs 15 ± 2 mm, *P* < .001). The coefficient of determination (*r*^2^) between LV-GLS and LA-GLS was .40 (95% CI 0.32–0.48) for the whole cohort, and 0.39 (95% CI 0.31–0.47) between LV-AVPD and LA-GLS.

**Conclusion:**

In a large cohort comprising both patients with LV dysfunction and HC, LA reservoir function quantified as LA-GLS was to a large extent determined by LV longitudinal function. LA function may not be an independent marker of global cardiac function for certain patient groups where diminished LA function can be a reflection of LV dysfunction.

## Graphical Abstract

**Graphical Abstract:**
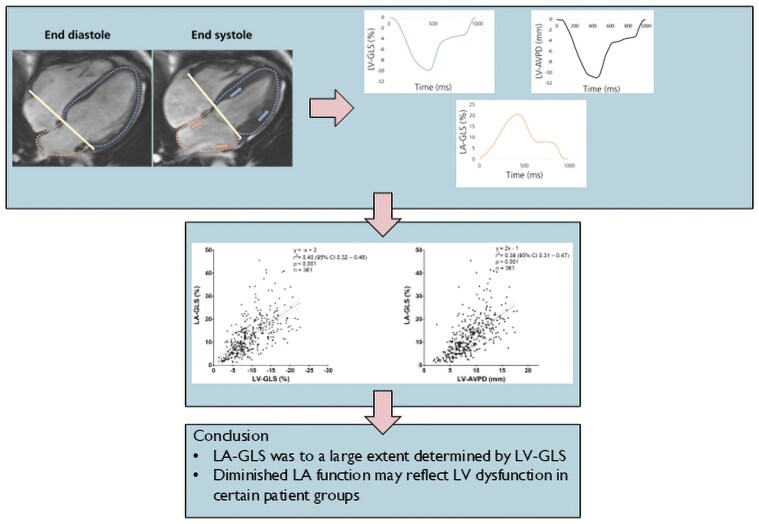
Association between left ventricular longitudinal function and left atrial strain from cardiac magnetic resonance in left ventricular dysfunction. LA: left atrial; LA-GLS: left atrial global longitudinal strain; LV: left ventricular; LV-AVPD: left ventricular atrioventricular plane displacement; LV-GLS: left ventricular global longitudinal strain.

## Introduction

Patients with heart failure (HF) with and without preserved ejection fraction (EF) have an increased risk of hospitalization and death.^1,2^ Left ventricular (LV) EF is routinely assessed using multiple imaging modalities and is a ubiquitous measure of ventricular dysfunction for clinical decision making and prognostication.^3^ Recently, atrial strain derived from cardiac magnetic resonance (CMR) has been proposed as a novel marker of outcome.^4,5^ However, the atria are physically connected to the ventricles by the fibrous atrioventricular plane and are therefore affected by ventricular longitudinal function.^6,7^ This physiological interaction between the left atrium (LA) and LV, often referred to as left atrioventricular coupling (LA–LV coupling),^8^ suggests an interdependence between the atria and ventricles. The association between LA and LV function has been explored using speckle-tracking echocardiography (STE), supporting moderate-to-strong LA–LV coupling,^9–11^ varying by phenotype. To the best of our knowledge, the degree to which LA function is affected by LV longitudinal function in patients with LV dysfunction has not been explored by CMR feature-tracking.

The aim of this study was therefore to quantify the association between LV and LA longitudinal function using CMR feature-tracking, in patients with different degrees of LV dysfunction, compared with healthy controls (HC).

## Methods

All study participants provided signed informed consent to participate in research, and the original studies were approved by the regional ethics committee in Lund, Sweden (Dnr 741/2004, Dnr 2010/380, Dnr 2011/668) and followed the Declaration of Helsinki. This retrospective, exploratory study included CMR examinations previously used in research,^12–15^ and complies with STROBE guidelines for reporting observational studies.^16^

### Study population

The study population was divided into the following groups: patients with HF with reduced EF (HFrEF), candidates for cardiac resynchronization therapy (CRT) implantation, ischaemic heart disease (IHD), and HC. Adult (≥18 years of age) patients with LV dysfunction who underwent CMR examination between 2003 and 2017 were included in the current study. Patients with HFrEF had a diagnosis of HF and LVEF ≤40%. Patients indicated for CRT fulfilled the class 1 recommendation according to guidelines.^3^ IHD was defined as first-time ST-elevation myocardial infarction (STEMI) with successful reperfusion after percutaneous coronary intervention and underwent CMR examination 6 months after intervention. HC with no previous known cardiovascular disease, normal blood pressure, and without ECG abnormalities were included as controls. Participants were excluded in the case of poor image quality, significant arrhythmia, including atrial fibrillation, or if the LA was not visible in a 4ch view. Complete inclusion and exclusion criteria were described in the respective study protocols^12–15^ (Appendix 1).

### Cardiac magnetic resonance imaging

CMR was performed on Philips Intera 1.5 T, Philips Achieva 3.0 T (Philips Medical System, Best, The Netherlands), or Siemens Aera 1.5 T scanner (Siemens Healthineers, Forchheim, Germany). Standard balanced steady-state free precession (bSSFP) cine images in short- and long-axis planes were acquired during end-expiratory breath-hold. Typical image parameters were as follows: temporal resolution: 30 ms; in-plane resolution: 1.5 × 1.5 mm^2^; slice thickness: 8 mm; slice gap: 0 mm; flip angle: 64°; echo time: 1.2 ms.

### Image analysis

Image analysis was performed using the freely available software Segment v4.0 R12067 (Medviso AB, Lund, Sweden, http://segment.heiberg.se).^17^ LV end-diastolic volume, end-systolic volume, and LV mass were calculated by delineating the endocardium and epicardium in short-axis images at end diastole and end systole. LV atrioventricular plane displacement (LV-AVPD) was measured as previously described.^18,19^ In brief, the atrioventricular plane was defined by eight input points that were manually marked in the 2-, 3-, and 4-chamber long-axis views in end diastole. An automatic algorithm tracked the input points through the cardiac cycle, and the input points were thereafter manually adjusted if needed. The mean perpendicular distance of the atrioventricular plane towards the apex in the three long-axis views is reported as LV-AVPD.

**Figure 1:**
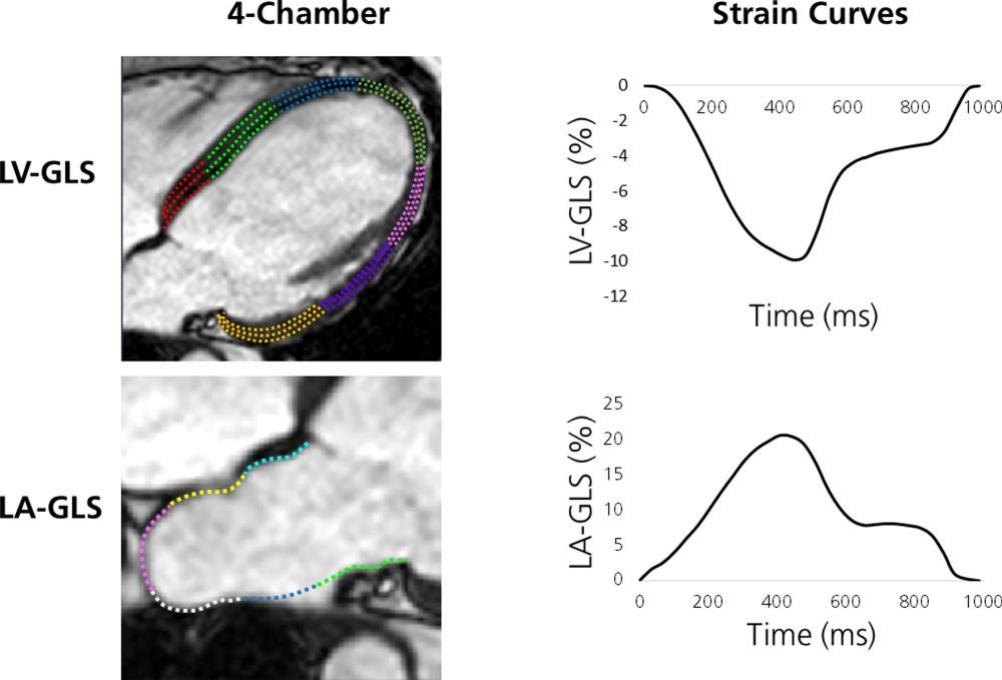
Example of left ventricular global longitudinal strain (LV-GLS) and left atrial GLS (LA-GLS) analysis. Top row: cardiac magnetic resonance (CMR) 4-chamber view with myocardial strain delineations superimposed (left) and LV-GLS curve during one cardiac cycle (right). Bottom row: CMR 4-chamber view with LA endocardial delineation superimposed (left) and LA-GLS curve during one cardiac cycle (right). Note that the two strain curves partly mirror each other

Feature-tracking myocardial strain was used to assess GLS in the LV and LA. LV-GLS was measured by end-diastolic delineation of the endocardium and epicardium in the 2-, 3-, and 4-chamber bSSFP cine images. LA-GLS was measured by delineation of the LA endocardial border in the 4-chamber view at ventricular end diastole. Atrial appendages and pulmonary veins were excluded from delineation. Delineations were automatically propagated by the algorithm throughout the cardiac cycle (*Figure 1*). In case of inadequate tracking, a new propagation was repeated after readjustments of the end-diastolic delineation. LA size was determined as the largest LA area from the propagated delineations.

### Statistical analysis

Statistical analyses were conducted using the software IBM SPSS version 25 (SPS inc., Chicago, Illinois, USA) and GraphPad Prism v10.0.2 for Windows (GraphPad Software, Boston, Massachusetts, USA). Continuous variables are expressed as means ± standard deviation or median and interquartile range according to normal distribution. Normal distribution was assessed graphically from histograms and *Q–Q* plots. Discrete variables are expressed as frequencies and proportions in percentages. Categorical data were compared using the Chi-square test. Patients with LV dysfunction (HFrEF, CRT, and IHD) were compared with HC using Student’s *t*-test for independent samples for continuous data. The four different study groups (HFrEF, CRT, IHD, and HC) were compared with each other using one-way ANOVA with *post hoc* Tukey’s HSD test for single dependent variables. Coefficient of determination (*r*^2^) analysis was conducted to analyse associations between LV longitudinal function (LV-GLS or LV-AVPD) and LA-GLS. Simple linear regression with interaction effect was used to compare group interaction regarding associations between LV longitudinal function and LA-GLS. Non-linear regression was conducted to investigate the relationship between LA area and LA-GLS, and between LA area and LV-GLS, using an exponential decay model. Univariable and multivariable linear regression analyses of LA-GLS were conducted to determine the independent effects of LV longitudinal function and confounders. Independent variables in the univariable linear regression analysis with a *P* value <.25 were included in the multivariable analysis. Intra- and inter-observer variability of LV-GLS and LA-GLS were assessed using Bland–Altman analysis and intraclass correlation coefficients in ten subjects. A two-tailed *P*-value of <.05 was defined as statistically significant.

## Results

Three hundred and sixty-one study participants (HFrEF, CRT, IHD, and HC) were included in the final data analysis, as eight were excluded due to poor image quality (*n* = 3), arrhythmia (*n* = 4), and LA not visible in 4ch view (*n* = 1). *Table 1* displays the characteristics of the study participants and Supplementary Table S1 displays available data on medication in the study participants. The patient group included a larger proportion of male participants and had a higher BMI and heart rate than HC. Patients had typical comorbidities and medication for a HF population.

**Table 1:** Characteristics of study participants

	HFrEF (*n* = 202)	CRT (*n* = 63)	IHD (*n* = 77)	All LV dysfunction (*n* = 342)	HC (*n* = 19)
Female	40 (20)	18 (29)	9 (12)	67 (20)*	8 (42)
Age (years)	60 ± 12	67 ± 9	59 ± 10	61 ± 12	63 ± 11
Height (cm)	175 ± 9	174 ± 9	176 ± 7	175 ± 9	173 ± 9
Weight (kg)	83 ± 16	85 ± 18	82 ± 11	83 ± 15*	75 ± 14
BMI (kg/m^2^)	26.8 ± 4.3	28.0 ± 5.2	26.7 ± 3.3	27.0 ± 4.3*	25.0 ± 3.2
BSA (m^2^)	1.98 ± 0.21	2.00 ± 0.22	1.98 ± 0.15	1.98 ± 0.20*	1.88 ± 0.21
HR (beats/min)	74 ± 16	68 ± 13	62 ± 10	70 ± 15***	62 ± 7
Morbidity					
History of IHD	118 (58)	32 (51)	77 (100)	227 (66)	n/a
HTN	52 (29)^a^	40 (64)	22 (29)	114 (36)^b^	n/a
DM	41 (23)^c^	9 (14)	8 (10)	58 (18)^d^	n/a

Categorical variables expressed as *n* (valid %) and continuous variables as mean ± SD.

BMI, body mass index; BSA, body surface area; CRT, candidates for cardiac resynchronization therapy; DM, diabetes mellitus; HC, healthy controls; HFrEF, heart failure with reduced ejection fraction; HR, heart rate; HTN, hypertension; IHD, iscahemic heart disease; LV, left ventricular.

*P < 0.05, **P < 0.01, ***P < 0.001 All LV dysfunction vs HC. a: *n* = 179, b: *n* = 319, c: *n* = 181, d = 322.

### Left ventricular size and function

**Figure 2:**
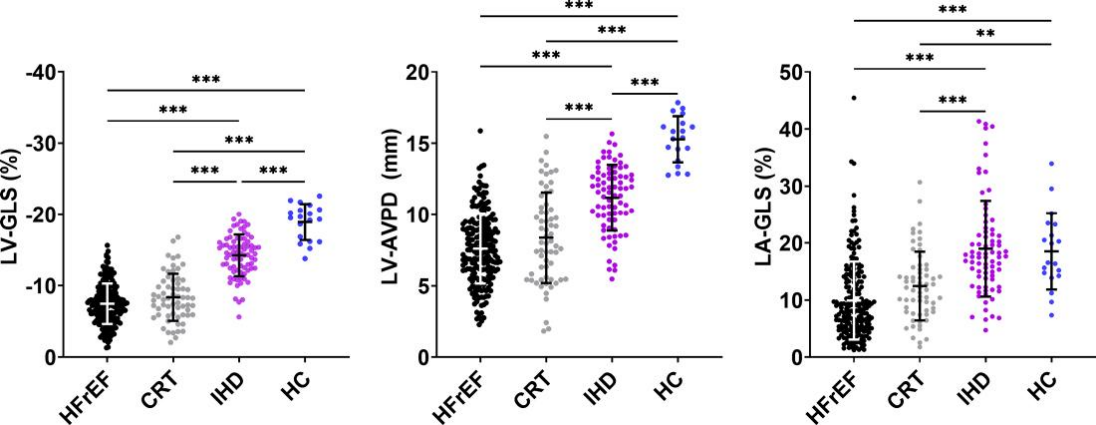
Left ventricular longitudinal and atrial function. Left: left ventricular global longitudinal strain (LV-GLS); Middle: left ventricular atrioventricular plane displacement (LV-AVPD). Right: left atrial global longitudinal strain (LA-GLS). Error bars denote mean ± SD. CRT: candidates for cardiac resynchronization therapy; HC: healthy controls; HFrEF: heart failure with reduced ejection fraction; IHD: ischaemic heart disease. *P < .05, **P < .01, ***P < .001

Left ventricular volumes were larger in patients with LV dysfunction (HFrEF, CRT, and IHD) than in HC (*Table 2*). Patients with LV dysfunction had impaired LV-GLS and LV-AVPD compared with HC (*Table 2*, *Figure 2*).

**Table 2:** Left ventricular and atrial size and longitudinal functional values from cardiac magnetic resonance imaging

	HFrEF (*n* = 202)	CRT (*n* = 63)	IHD (*n* = 77)	All LV dysfunction (*n* = 342)	HC (*n* = 19)
Left ventricle					
LVEDV (ml)	298 ± 92	327 ± 115	190 ± 43	279 ± 101***	161 ± 38
LVESV (ml)	224 ± 87	248 ± 110	93 ± 35	199 ±101***	65 ± 21
LVEF (%)	26 ± 8	26 ± 8	52 ± 10	32 ± 14***	60 ± 5
LV-GLS (%)	−7 ± 3	−8 ± 3	−14 ± 3	−9 ± 4***	−19 ± 3
LV-AVPD (mm)	8 ± 2	8 ± 3	11 ± 2	9 ± 3***	15 ± 2
Left atrium					
LA area (cm^2^)	31 ± 8	28 ± 7	23 ± 5	28 ± 8*	26 ± 3
LA-GLS (%)	10 ± 7	12 ± 6	19 ± 8	12 ± 8***	19 ± 7

Continuous variables are expressed as mean ± SD.

AVPD, atrioventricular plane displacement; CRT, candidates for cardiac resynchronization therapy; EDV, end-diastolic volume; ESV, end-systolic volume; EF, ejection fraction; GLS, global longitudinal strain; HC, healthy controls; HFrEF, heart failure with reduced ejection fraction; IHD, ischaemic heart disease; LA, left atrial; LV, left ventricular.

*P < .05, **P < .01, ***P < .001 All LV dysfunction vs HC.

LV-GLS differed among the study groups, except for HFrEF vs CRT (*P* = .15). Similarly, LV-AVPD differed among groups except for HFrEF vs CRT (*P* = .16) (*Table 2*, *Figure 2*).

### Left atrial size and function

Patients with LV dysfunction had lower LA-GLS compared with HC (*Table 2*, *Figure 2*). LA-GLS differed among the four study groups, except for HFrEF vs CRT (*P* = .06) and IHD vs HC (*P* = .99) (*Table 2*, *Figure 2*).

**Figure 3:**
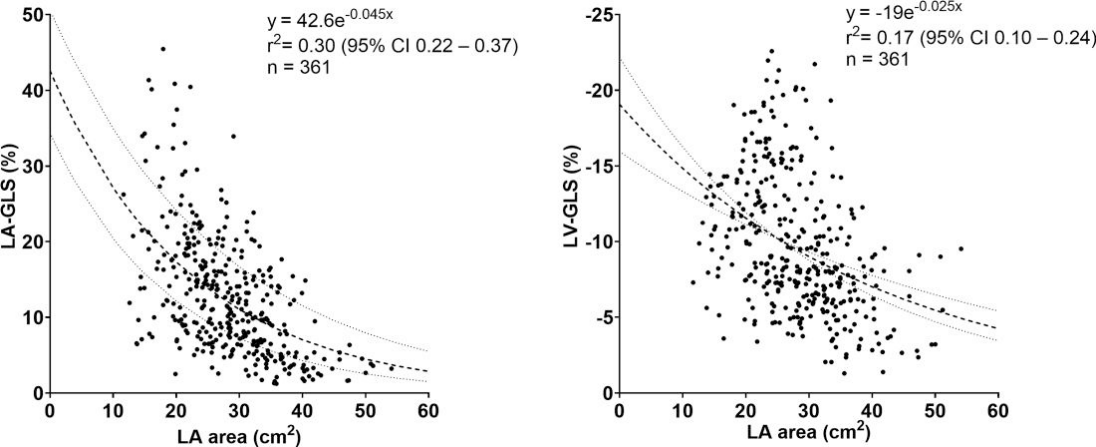
Relationship between left atrial (LA) area and LA global longitudinal strain (GLS) to the left, and between LA area and left ventricular (LV) GLS to the right, in the whole study population. The regression lines with 95% confidence bands are indicated with dashed lines

Patients with LV dysfunction had a larger LA area compared with HC (*Table 2*, *Figure 2*). Non-linear regressions of the relationships between LA area and LA-GLS, and between LA area and LV-GLS are depicted in *Figure 3*. The *r*^2^ value of the non-linear relationship between LA area and LA-GLS was 0.30 (95% CI 0.22–0.37), and the *r*^2^ value between LA area and LV-GLS was 0.17 (95% CI 0.10–0.24), indicating that LA area explains 30% of the variance in LA-GLS and 17% of the variance in LV-GLS according to the exponential decay models.

### Association between ventricular and atrial function

**Figure 4:**
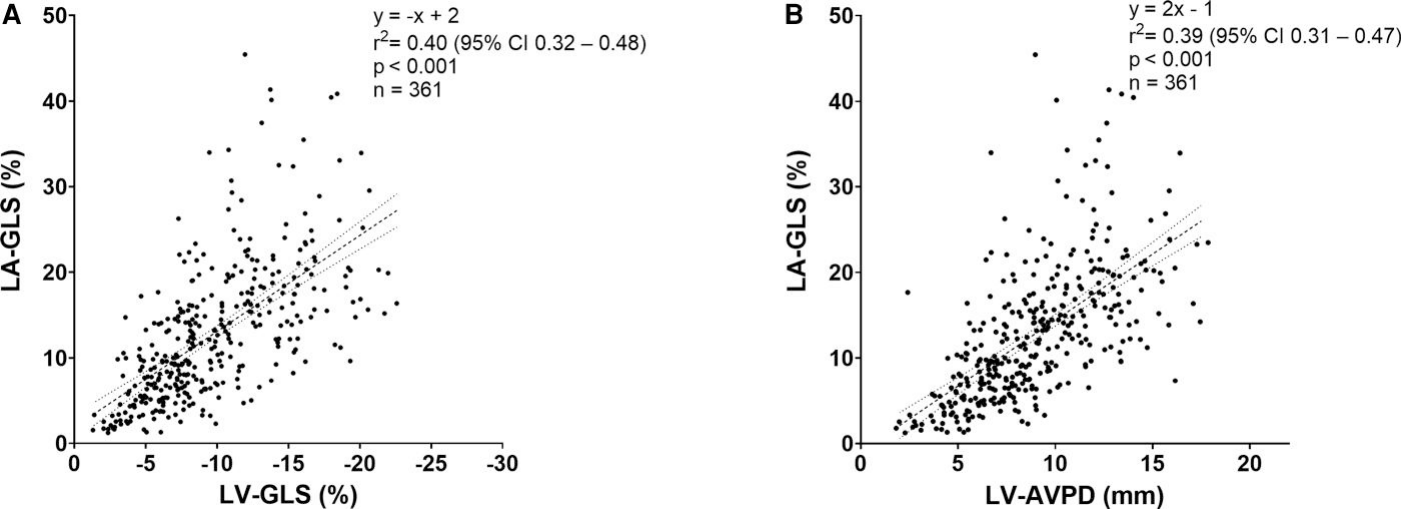
Relationship between left ventricular and atrial function in the whole study cohort. *A*: correlation between left ventricular global longitudinal strain (LV-GLS) and left atrial GLS (LA-GLS). *B*: correlation between LV atrioventricular plane displacement (LV-AVPD) and LA-GLS). The regression lines with 95% confidence bands are indicated with dashed lines

A linear association between LV-GLS and LA-GLS, and LV-AVPD and LA-GLS in the whole study cohort is shown in *Figure 4*. LV longitudinal function determined about 40% of the variance in LA-GLS in the whole study population. Univariable and multivariable analyses in the whole study population showed that LVEF and IHD are independently associated with LA-GLS when adjusting for confounders (*P* < .05, *Table 3*).

**Table 3:** Univariable and multivariable linear regression analysis of left atrial global longitudinal function in the whole cohort (*n* = 361)

	Dependent variable: LA-GLS			
	Univariable		Multivariable	
	β	*P*	β	*P*
LVEDV	−0.03	*<*.*001*	0.003	.72
LV-GLS	−1.12	*<*.*001*	−0.07	.84
LVEF	0.30	*<*.*001*	0.32	.*01*
LA area	−0.58	*<*.*001*	−0.09	.47
HR	−0.18	*<*.*001*	0.07	.90
SBP	0.08	.*08*	0.08	.05
Age	−0.03	.49		
Sex	2.63	.*01*	2.27	.14
BMI	−0.004	.97		
IHD	1.26	.*17*	−3.67	.*02*
HTN	0.17	.85		
DM	−1.71	.*05*	−1.12	.17

AVPD, atrioventricular plane displacement; BMI, body mass index; DM, diabetes mellitus; EDV, end-diastolic volume; GLS, global longitudinal strain; HR, heart rate; HTN, hypertension; IHD, ischaemic heart disease; LA, left atrial; LV, left ventricular; SBP, systolic blood pressure. *P*-values in italics indicate statistical significance (*P* < .25 for univariable analysis; *P* < .05 for multivariable analysis).

**Figure 5:**
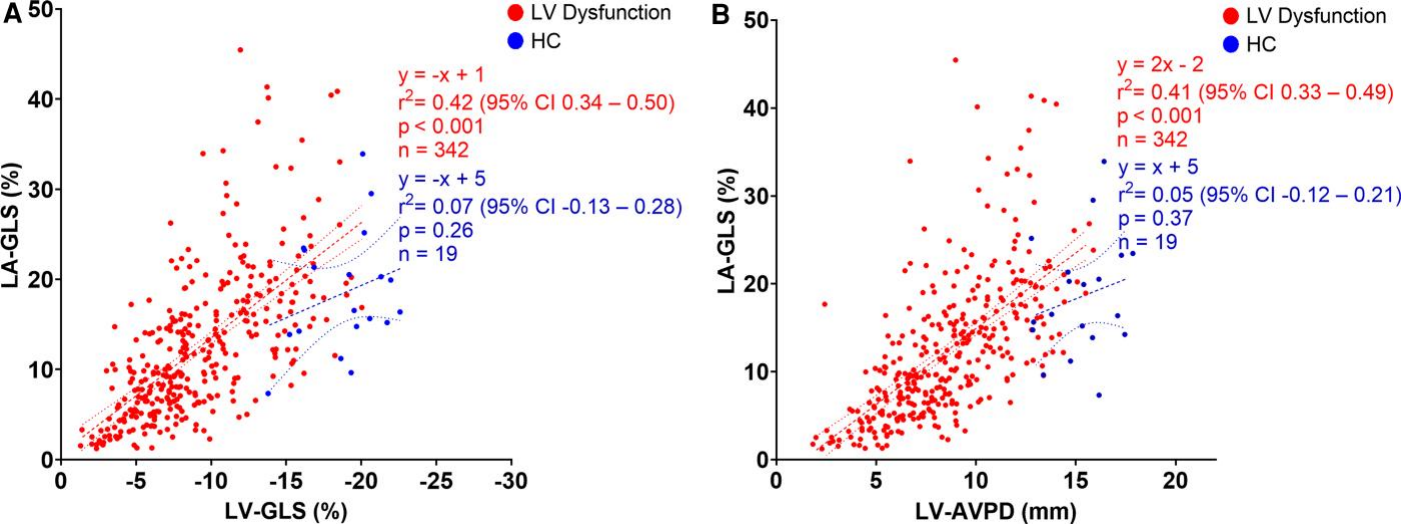
Relationship between left ventricular and atrial function in patients with left ventricular (LV) dysfunction (HFrEF, CRT, and IHD; red) and healthy controls (HC; blue) separated. *A*: correlation between LV global longitudinal strain (LV-GLS) and left atrial GLS (LA-GLS). *B*: correlation between LV atrioventricular plane displacement (LV-AVPD) and LA-GLS). The regression lines with 95% confidence bands are indicated with dashed lines

When comparing patients with LV dysfunction to HC, 42% (95% CI 34%—50%) of the variance in LA-GLS was determined by LV-GLS in patients with LV dysfunction, with an inconclusive association (*P* = .26) in HC (*Figure 5*). Regarding LV-AVPD, there was a similar association to LA-GLS in patients with LV dysfunction (*r*^2^ = 0.41, 95% CI 0.33–0.49), yet an inconclusive association in HC (*P* = .37) (*Figure 5*). Simple linear regression with group interaction was not statistically significant for LV-GLS (*P* = .33) nor LV-AVPD (*P* = .36), indicating that the association between LV longitudinal function and LA-GLS did not conclusively differ between patients with LV dysfunction and HC.

**Figure 6:**
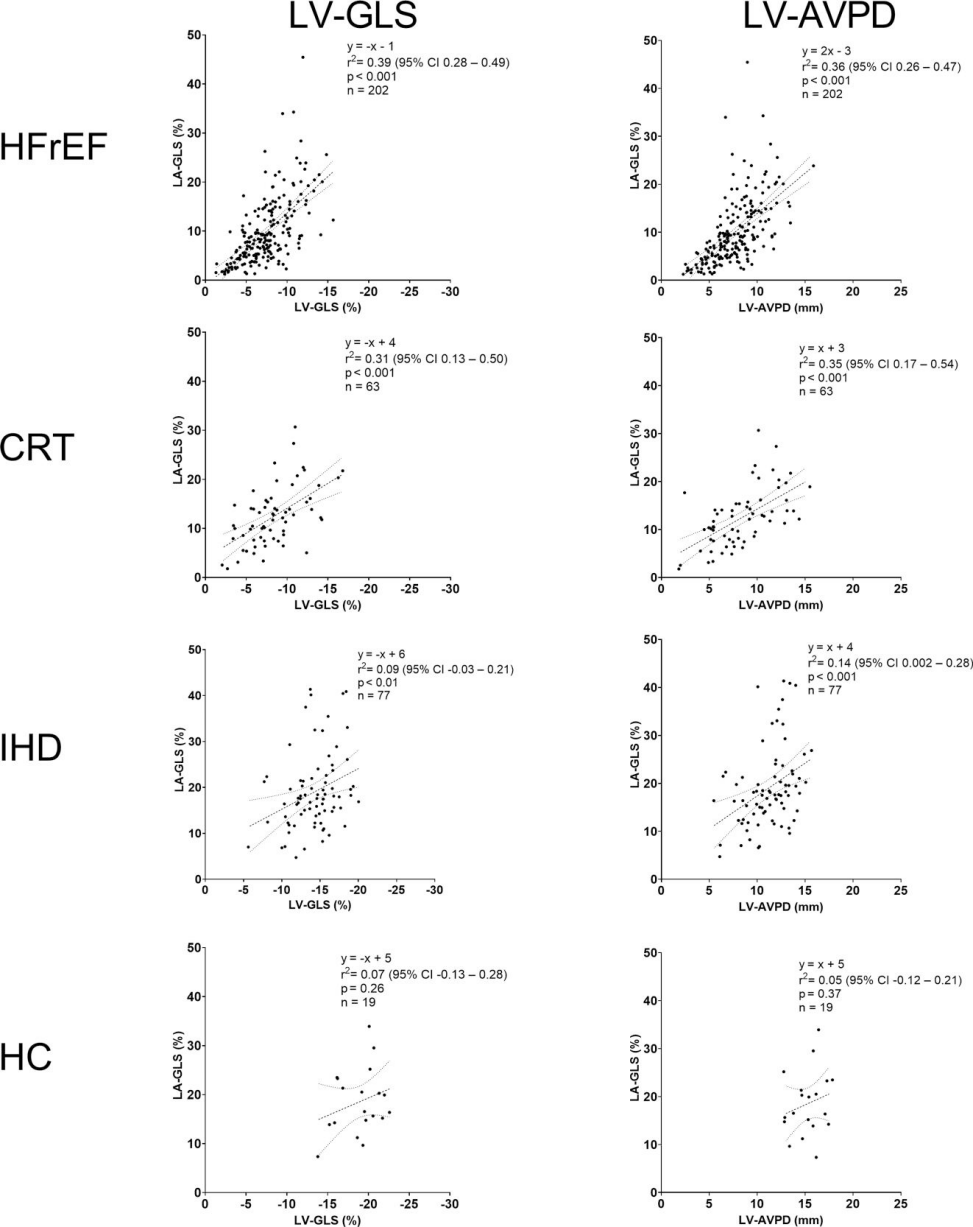
Association between left ventricular global longitudinal strain (LV-GLS) and left atrial GLS (LA-GLS) (left column) and left ventricular atrioventricular plane displacement (LV-AVPD) and LA-GLS (right column) in study groups. The regression lines are indicated with dashed lines. CRT: candidates for cardiac resynchronization therapy; HC: healthy controls; HFrEF: heart failure with reduced ejection fraction; IHD: ischaemic heart disease

On a subgroup level, comparing the four study groups with each other, LA-GLS was determined by both LV-GLS and LV-AVPD in HFrEF, CRT, and IHD, but not for HC (*Figure 6*). Simple linear regression with group interaction was not statistically significant for LV-GLS (*P* = .08) nor LV-AVPD (*P* = .26), indicating that the association between LV longitudinal function and LA-GLS did not conclusively differ between groups. Additionally, subgroup analysis was done dividing study participants into the following groups: LVEF < median vs LVEF ≥ median, hypertension vs normotension, and diabetes mellitus vs non-diabetes mellitus (Supplementary Figure S1). Simple linear regression with group interaction was statistically significant in the LVEF < median vs LVEF ≥ median analysis for LV-GLS (*P* = .016), indicating that the association between LV-GLS and LA-GLS is dependent on LVEF. There was a stronger relationship between LV-GLS and LA-GLS in the group with LVEF < median (*r*^2^ = 0.29, 95% CI 0.18–0.40) compared with the group with LVEF ≥ median (*r*^2^ = 0.18, 95% CI 0.08–0.28).

### Intra- and inter-observer variability

Intra- and inter-observer analyses of LV-GLS and LA-GLS showed excellent reliability in measurements with ICC >0.96 for the measurements. Absolute biases for intra-observer variability were ≤1% and <4% for LV-GLS and LA-GLS, respectively. Inter-observer variability was <8% for LV-GLS and <14% for LA-GLS (Supplementary Table S2, Supplementary Figures S2 and S3).

## Discussion

In this large, heterogeneous, real-world population comprising HC and patients presenting with varying grades of LV dysfunction, LA function was to a large extent determined by LV longitudinal function. In the patient group alone, just above 40% of LA function was determined by LV longitudinal function, with large variations among subgroups. In HC, LA function was not significantly associated with LV longitudinal function. LA size, however, for the whole study population, can explain 30% of LA function. This CMR feature-tracking study demonstrates LA–LV coupling in a large cohort of patients with varying degrees of LV dysfunction and HC. Hence, LA function may not offer additional diagnostic or prognostic value to LV longitudinal function for certain patient groups when assessed in isolation.

### Clinical significance

LVEF is routinely utilized as a measure of cardiac function during clinical decision-making.^20^ However, due to high variability, modest reproducibility, and limited ability to assess preclinical dysfunction, more sophisticated measures of cardiac function are necessary. LA-GLS has been shown in isolation to be a prognostic marker for mortality and hospitalization,^4,5^ albeit using a simplified method of LA longitudinal function. The results from this study using the more detailed method of feature-tracking strain indicate that the association between LV longitudinal function, expressed as LV-GLS and LV-AVPD, and LA-GLS is not the same in all patient populations. Even among the patients with LV dysfunction in the present study, the explanatory value between LV-GLS or LV-AVPD and LA-GLS ranged from 9% to 39%. This suggests that LA-GLS is partly driven by LV longitudinal function, but to varying degrees in different patient populations depending on aetiology and LV function.

Patients with LV dysfunction had impaired LV volume and function compared with HC. However, when comparing the three subgroups of patients with LV dysfunction, IHD was less affected in LV volume and function than HFrEF and CRT, with less dilated LVEDV and better LVEF and LV-GLS, albeit with a mildly reduced LV systolic function justifying representation in the LV dysfunction cohort. Furthermore, IHD had preserved LA function with LA-GLS similar to HC (*P* = .99), in contrast to HFrEF and CRT who had impaired LA function with decreased LA-GLS compared with HC. Previous studies on patients with STEMI have reported preserved LA function at follow-up when atrial remodelling is absent, but diminished LA function in the case of atrial enlargement.^21,22^ It can be hypothesized from this observation that LA function is preserved for longer and does not decrease in parallel as LV function deteriorates in IHD. The point where LA function starts to decrease may be of important prognostic value, and it could be speculated that the mechanism of LA function impairment may be partially independent of LV function and differ between patient groups. For instance, the STE-derived LA strain has been shown to be impaired before LA and LV remodelling, and LV dysfunction in patients with hypertension.^23^

In general, stronger associations between LV and LA function were observed within larger study groups, encompassing both the entire study population and all patients with LV dysfunction. When considering all study participants irrespective of LV function, the explanatory value between LV-GLS or LV-AVPD and LA-GLS ranged from 39% to 40%. Conversely, HC did not demonstrate a linear relationship between LV longitudinal function (neither GLS nor AVPD) and LA-GLS. This discrepant observation between patients with LV dysfunction and HC may be explained by a small variance in LV-GLS in HC. Thus, it can be speculated that when LV longitudinal function is diminished due to pathology, LA dysfunction follows, indicating a close relationship.

The statistical analysis of the study groups regarding the relationship between LV longitudinal function and LA function did not reveal significant differences, apart from a subgroup analysis of study participants with LVEF < median vs LVEF ≥ median. These results from the present study combined with findings from previous studies showing differences in LA–LV coupling across phenotypes, e.g. in hypertrophic cardiomyopathy^24^ and dilated cardiomyopathy,^25^ suggest that LV function has a relatively stronger influence on LA function, and thereby LA–LV coupling, compared with other pathological changes of the LA and LV, e.g. fibrosis and compliance.

The findings from the present CMR study are in line with previous STE studies exploring the association between LA and LV function, indicating a significant but not universal LV-driven determinant of LA strain.^9–11^ Methodologically, CMR-FT measures myocardial borders while STE tracks myocardial speckles. The methods are not interchangeable due to modest correlation and suboptimal agreement,^26^ and they have inherent differences in spatial and temporal resolution. Thus, in addition to phenotype, modality may influence the correlation between LA and LV functions.

### Left ventricular longitudinal function

Length deformation in the longitudinal plane can be measured either in relative terms, such as LV-GLS, or in absolute terms as LV-AVPD. As the LA and LV have a common atrioventricular plane, it could be hypothesized that LV dysfunction with impaired LV longitudinal function would have a direct effect on LA-GLS. The results from the present study support this hypothesis, as ∼40% of the variation in LA-GLS was explained by LV-GLS and LV-AVPD in the whole cohort, and there was a positive correlation between LV-GLS or LV-AVPD and LA-GLS.

LA-GLS was to a large degree dependent on atrioventricular structural mechanics as there was an independent association with LV-GLS after adjusting for confounders. However, LA function was not fully explained by LV longitudinal function when analysing the explanatory values. The remaining explanatory variables need to be determined in future studies, but it could be suggested that atrial contractility, preload, and afterload each contribute to the variation in LA-GLS.

### Cardiac loading conditions

Beta-adrenergic antagonists, calcium channel antagonists, diuretics, and medications targeting the renin-angiotensin-aldosterone system are medications known to lower pre- and afterload conditions of the heart. Unfortunately, data on medication were not available for all study participants and may have affected the results since GLS is a load-dependent measure of myocardial function.^27^ It is expected that a reduction in afterload has a positive effect on GLS and LV-AVPD, while a reduction in preload has a negative effect.^28^ On the same note, increased heart rate affects preload by reducing filling time,^29^ and that could be affecting GLS negatively.^30^ Heart rate differed amongst groups, and increased heart rate may, in part, lower LV-GLS and LA-GLS as seen in patients with LV dysfunction compared with HC.

In the current study, 33% of patients with LV dysfunction had hypertension. Hypertension is a state of increased systemic vascular resistance and thereby increased myocardial wall tension during systole. This elevated afterload can be propagated back towards the LA and hypertension therefore has a negative effect on LA-GLS,^31^ causing alterations in LA–LV coupling.^32^ Furthermore, 17% of patients had a history of diabetes and 66% of IHD, both affecting strain. Diabetes has independently been associated with reduced LA strain^33^ and IHD affects LV-GLS.^34^ Thus, medication and cardiovascular risk factors in the present study may interact, affecting preload and afterload and thereby influencing LV- and LA-GLS.

### Limitations

Some limitations of this study need to be noted. The retrospective, single-centre study design introduces potential risks for selection bias, and the conclusions drawn from the findings may only be hypothesis-generating for future, prospective studies.

Data acquisition from study participants used different vendors (Philips and Siemens) with different magnetic field strengths (1.5 and 3.0 T), introducing technical heterogeneity in the data acquisition phase of the study. Thus, the comparability of images may be affected.^35^ However, it should be emphasized that the data analysis of volumes and function, including myocardial feature-tracking strain analysis, was performed in the same version of the image analysis software and intra- and inter-observer analysis showed excellent reliability in measurements.

Data on medications affecting preload and afterload, such as beta-adrenergic antagonists, renin-angiotensin-aldosterone system inhibitors, calcium channel antagonists, and diuretics, were not available for all patients. These medications may influence atrial and ventricular parameters and would ideally be included among the other confounding factors in the analyses.

Study groups differed in demographic and cardiovascular risk factors that may confound the results of this observational study, e.g. age, sex, history of hypertension, diabetes, and IHD. However, the association between LV and LA longitudinal function remained even after adjusting for confounders. The group of LV dysfunction consisted of more men compared with the HC group, echoing the known problem of unequal representation between sexes in cardiovascular research.^36^ The generalizability is limited as some cardiovascular disorders and risk factors, such as arrhythmia were not represented in the study population.

The heterogeneous group of patients with LV dysfunction (HFrEF, CRT, and IHD) consisted of three subgroups with different pathophysiology, introducing variation in the degree and duration of LV dysfunction. This may in turn affect atrial and ventricular strain to varying degrees, even in the same subgroup of patients with LV dysfunction. Future studies may implement strict group stratification to achieve homogenous groups and thereby reduce variance. Stricter group stratification may additionally enable investigation of the effect of phenotype-specific pathophysiological changes, e.g. fibrosis and compliance, on LA–LV coupling.

Study groups were unbalanced in sample sizes, ranging from *n* = 19 (HC) to *n* = 202 (HFrEF), primarily affecting statistical power and variance. Results, especially when comparing patients with LV dysfunction with HC, should consequently be interpreted with caution.

## Conclusion

About 40% of LA function was most likely determined by LV longitudinal function in this large, heterogeneous patient cohort with LV dysfunction, as well as in HC. The LA–LV coupling was less pronounced within specific subgroups, yet these results suggest LA function is dependent on LV longitudinal function. Furthermore, LV and LA size, and LV longitudinal function were independently associated with LA-GLS (reservoir strain). Consequently, LA function may not be an independent marker of cardiac health for certain patient groups where a loss of LA function is a reflection of LV dysfunction.

## Supplementary Material

xvag046_Supplementary_Data

## Declarations

### Data Availability

No data were generated or analysed for this manuscript.

### Pre-registered Clinical Trial Number

None supplied.

## References

[1] Jackson SL, Tong X, King RJ, Loustalot F, Hong Y, Ritchey MD. National burden of heart failure events in the United States, 2006 to 2014. Circ Heart Fail 2018;11:e004873. 10.1161/CIRCHEARTFAILURE.117.004873 30562099 PMC6424109

[2] Carson PE, Anand IS, Win S, Rector T, Haass M, Lopez-Sendon J, et al The hospitalization burden and post-hospitalization mortality risk in heart failure with preserved ejection fraction: results from the I-PRESERVE trial (irbesartan in heart failure and preserved ejection fraction. JACC Heart Fail 2015;3:429–41. 10.1016/j.jchf.2014.12.017 25982110

[3] Ponikowski P, Voors AA, Anker SD, Bueno H, Cleland JGF, Coats AJS, et al 2016 ESC Guidelines for the diagnosis and treatment of acute and chronic heart failure. Eur Heart J 2016;37:2129–200. 10.1002/ejhf.592. Epub 2016 27206819

[4] Leng S, Ge H, He J, Kong L, Yang Y, Yan F, et al Long-term prognostic value of cardiac MRI left atrial strain in ST-segment elevation myocardial infarction. Radiology 2020;296:299–309. 10.1148/radiol.2020200176 32544032

[5] Li Y, Xu Y, Tang S, Jiang X, Li W, Guo J, et al Left atrial function predicts outcome in dilated cardiomyopathy: fast long-axis strain analysis derived from MRI. Radiology 2022;302:72–81. 10.1148/radiol.2021210801 34698565

[6] Barbier P, Solomon SB, Schiller NB, Glantz SA. Left atrial relaxation and left ventricular systolic function determine left atrial reservoir function. Circulation 1999;100:427–36. 10.1161/01.cir.100.4.427 10421605

[7] Bowman AW, Kovács SJ. Left atrial conduit volume is generated by deviation from the constant-volume state of the left heart: a combined MRI-echocardiographic study. Am J Physiol Heart Circ Physiol 2004;286:2416–24. 10.1152/ajpheart.00969.2003 14751859

[8] Pezel T, Venkatesh BA, De Vasconcellos HD, Kato Y, Shabani M, Xie E, et al Left atrioventricular coupling index as a prognostic marker of cardiovascular events: the MESA study. Hypertension 2021;78:661–71. 10.1161/HYPERTENSIONAHA.121.17339 34225471 PMC8363553

[9] Mălăescu GG, Mirea O, Capotă R, Petrescu AM, Duchenne J, Voigt JU. Left atrial strain determinants during the cardiac phases. JACC Cardiovasc Imaging 2022;15:381–91. 10.1016/j.jcmg.2021.09.009 34656486

[10] Nishikage T, Yamamoto H, Fukumoto N, Takahashi K, Ota Y, Kusaki H, et al Significant dependency of left atrial strain on left ventricular longitudinal motion. J Echocardiogr 2023;21:149–56. 10.1007/s12574-023-00605-z 37261702

[11] Kupczyńska K, Mandoli GE, Cameli M, Kasprzak JD. Left atrial strain—A current clinical perspective. Kardiol Pol 2021;79:955–64. 10.33963/KP.a2021.0105 34599503

[12] Berg J, Jablonowski R, Mohammad M, Solem K, Borgquist R, Ostenfeld E, et al Ventricular longitudinal shortening is an independent predictor of death in heart failure patients with reduced ejection fraction. Sci Rep 2021;11:1–13. 10.1038/s41598-021-99613-1 34645886 PMC8514526

[13] Borgquist R, Carlsson M, Markstad H, Werther-Evaldsson A, Ostenfeld E, Roijer A, et al Cardiac resynchronization therapy guided by echocardiography, MRI, and CT imaging: a randomized controlled study. JACC Clin Electrophysiol 2020;6:1300–9. 10.1016/j.jacep.2020.05.011 33092758

[14] Erlinge D, Götberg M, Lang I, Holzer M, Noc M, Clemmensen P, et al Rapid endovascular catheter core cooling combined with cold saline as an adjunct to percutaneous coronary intervention for the treatment of acute myocardial infarction: the CHILL-MI trial: a randomized controlled study of the use of central venous catheter core cooling combined with cold saline as an adjunct to percutaneous coronary intervention for the treatment of acute myocardial infarction. J Am Coll Cardiol 2014;63:1857–65. 10.1016/j.jacc.2013.12.027 24509284

[15] Asgeirsson D, Hedström E, Jögi J, Pahlm U, Steding-Ehrenborg K, Engblom H, et al Longitudinal shortening remains the principal component of left ventricular pumping in patients with chronic myocardial infarction even when the absolute atrioventricular plane displacement is decreased. BMC Cardiovasc Disord 2017;17:1–9. 10.1186/s12872-017-0641-z 28754098 PMC5534092

[16] Vandenbroucke JP, Von Elm E, Altman DG, Gøtzsche PC, Mulrow CD, Pocock SJ, et al Strengthening the reporting of observational studies in epidemiology (STROBE): explanation and elaboration. Epidemiology 2007;4:1628–54. 10.1097/EDE.0b013e3181577511 18049195

[17] Heiberg E, Sjögren J, Ugander M, Carlsson M, Engblom H, Arheden H. Design and validation of segment–freely available software for cardiovascular image analysis. BMC Med Imaging 2010;10:1. 10.1186/1471-2342-10-1 20064248 PMC2822815

[18] Carlsson M, Ugander M, Mosén H, Buhre T, Arheden H. Atrioventricular plane displacement is the major contributor to left ventricular pumping in healthy adults, athletes, and patients with dilated cardiomyopathy. Am J Physiol Heart Circ Physiol 2007;292:H1452–9. 10.1152/ajpheart.01148.2006 17098822

[19] Seemann F, Pahlm U, Steding-Ehrenborg K, Ostenfeld E, Erlinge D, Dubois-Rande JL, et al Time-resolved tracking of the atrioventricular plane displacement in cardiovascular magnetic resonance (CMR) images. BMC Med Imaging 2017;17:1–16. 10.1186/s12880-017-0189-5 28241751 PMC5330030

[20] Knuuti J, Wijns W, Saraste A, Capodanno D, Barbato E, Funck-Brentano C, et al 2019 ESC Guidelines for the diagnosis and management of chronic coronary syndromes. Eur Heart J 2020;41:407–77. 10.1093/eurheartj/ehz425 31504439

[21] Attanasio A, Tondi L, Castelvecchio S, Pazzanese V, Palmisano A, Esposito A, et al Left atrial dysfunction predicts left ventricular remodelling in patients with preserved ejection fraction after acute ST-elevation myocardial infarction. Eur J Prev Cardiol 2024 Feb 21. 10.1093/eurjpc/zwae072 38381565

[22] Antoni ML, Brinke T, Marsan EA, Atary NA, Holman JZ, Van Der Wall ER, et al Comprehensive assessment of changes in left atrial volumes and function after ST-segment elevation acute myocardial infarction: role of two-dimensional speckle-tracking strain imaging. J Am Soc Echocardiogr 2011;24:1126–33. 10.1016/j.echo.2011.06.017 21820865

[23] Cameli M, Ciccone MM, Maiello M, Modesti PA, Muiesan ML, Scicchitano P, et al Speckle tracking analysis: a new tool for left atrial function analysis in systemic hypertension: an overview. J Cardiovasc Med 2016;17:339–43. 10.2459/JCM.0000000000000073 24838034

[24] De Raffele M, Teis A, Cediel G, Weerts J, Conte C, Juncà G, et al Left atrial remodelling and function in various left ventricular hypertrophic phenotypes. Eur Heart J Cardiovasc Imaging 2025;26:853–62. 10.1093/ehjci/jeaf033 39874262

[25] Zhou D, Wang Y, Li S, Wu W, Sun X, Zhuang B, et al Ventricular-atrial coupling in subjects with normal, preserved, and reduced left ventricular ejection fraction: insights from cardiac magnetic resonance imaging. Eur Radiol 2023;33:7716–28. 10.1007/s00330-023-09801-y 37318603

[26] Pryds K, Larsen AH, Hansen MS, Grøndal AYK, Tougaard RS, Hansson NH, et al Myocardial strain assessed by feature tracking cardiac magnetic resonance in patients with a variety of cardiovascular diseases—a comparison with echocardiography. Sci Rep 2019;9:11296. 10.1038/s41598-019-47775-4 31383914 PMC6683180

[27] Dahle GO, Stangeland L, Moen CA, Salminen PR, Haaverstad R, Matre K, et al The influence of acute unloading on left ventricular strain and strain rate by speckle tracking echocardiography in a porcine model. Am J Physiol Heart Circ Physiol 2016;310:H1330–9. 10.1152/ajpheart.00947.2015 26968547 PMC4895836

[28] Genovese D, Singh A, Volpato V, Kruse E, Weinert L, Yamat M, et al Load dependency of left atrial strain in normal subjects. J Am Soc Echocardiogr 2018;31:1221–8. 10.1016/j.echo.2018.07.016 30205909 PMC7147871

[29] Chung CS, Karamanoglu M, Kovács SJ. Duration of diastole and its phases as a function of heart rate during supine bicycle exercise. Am J Physiol Heart Circ Physiol 2004;287:2003–8. 10.1152/ajpheart.00404.2004 15217800

[30] Weidemann F, Jamal F, Sutherland GR, Claus P, Kowalski M, Hatle L, et al Myocardial function defined by strain rate and strain during alterations in inotropic states and heart rate. Am J Physiol Heart Circ Physiol 2002;283:H792–9. 10.1152/ajpheart.00025.2002 12124229

[31] Burns AT, La Gerche A, D’hooge J, Macisaac AI, Prior DL. Left ventricular strain and strain rate: characterization of the effect of load in human subjects. Eur J Echocardiogr 2010;11:283–9. 10.1093/ejechocard/jep214 20026455

[32] Shi R, Jiang YN, Qian WL, Guo YK, Gao Y, Shen LT, et al Assessment of left atrioventricular coupling and left atrial function impairment in diabetes with and without hypertension using CMR feature tracking. Cardiovasc Diabetol 2023;22:295. 10.1186/s12933-023-01997-z 37904206 PMC10617180

[33] Shen MT, Guo YK, Liu X, Ren Y, Jiang L, Xie LJ, et al Impact of BMI on left atrial strain and abnormal atrioventricular interaction in patients with type 2 diabetes Mellitus: a cardiac magnetic resonance feature tracking study. J Magn Reson Imaging 2022;55:1461–75. 10.1002/jmri.27931 34549860

[34] Rost C, Rost MC, Breithardt OA, Schmid M, Klinghammer L, Stumpf C, et al Relation of functional echocardiographic parameters to infarct scar transmurality by magnetic resonance imaging. J Am Soc Echocardiogr 2014;27:767–74. 10.1016/j.echo.2014.02.004 24651003

[35] Yang W, Xu J, Zhu L, Zhang Q, Wang Y, Zhao S, et al Myocardial strain measurements derived from MR feature-tracking: influence of sex, age, field strength, and vendor. JACC Cardiovasc Imaging 2024;17:364–79. 10.1016/j.jcmg.2023.05.019 37480906

[36] Bucciarelli-Ducci C, Ostenfeld E, Baldassarre LA, Ferreira VM, Frank L, Kallianos K, et al Cardiovascular disease in women: insights from magnetic resonance imaging. J Cardiovasc Magn Reson 2020;22:71. 10.1186/s12968-020-00666-4 32981527 PMC7520984

