# Supplementary material for: Association between left ventricular longitudinal function and left atrial strain in left ventricular dysfunction
Source: ESC Heart Fail. 2026 Feb 10;13(2):xvag046. doi: 10.1093/eschf/xvag046 (PMC12988773; doi:10.1093/eschf/xvag046)
Supplement: xvag046_Supplementary_Data [file xvag046_supplementary_data.zip › Appendix 1.docx]

# Appendix 1

# Inclusion and Exclusion Criteria for Study Populations

## Heart Failure with Reduced Ejection Fraction

**Inclusion Criteria**

- Clinical diagnosis of heart failure with reduced ejection fraction (HFrEF)
- Clinical cardiac magnetic resonance (CMR) imaging examination at Skåne University Hospital in Lund during 2003 – 2015
- Prior to CMR informed written consent to have data used for research purposes

**Exclusion Criteria**

- Left ventricular ejection fraction >40% measured from the CMR examination
- Inadequate image quality

**Reference**

Berg J, Jablonowski R, Mohammad M, Solem K, Borgquist R, Ostenfeld E, et al. Ventricular longitudinal shortening is an independent predictor of death in heart failure patients with reduced ejection fraction. 2021;11(1):1–13.

## Cardiac Resynchronization Therapy

**Inclusion Criteria**

- Referred to the arrhythmia section at Skåne University Hospital in Lund for cardiac resyncronization therapy (CRT) implantation during 2012 – 2017
- Fulfilled guideline class I indications for CRT
- Adequate image quality from baseline echocardiography examinations

**Exclusion Criteria**

- Permanent atrial fibrillation
- Pregnancy
- Adult congenital heart disease
- Severe renal failure

**Reference**

Borgquist R, Carlsson M, Markstad H, Werther-Evaldsson A, Ostenfeld E, Roijer A, et al. Cardiac Resynchronization Therapy Guided by Echocardiography, MRI, and CT Imaging: A Randomized Controlled Study. 2020;6(10):1300–9.

## Ischemic Heart Disease

**Inclusion Criteria**

- Age 18 – 75 years
- Presenting with anterior or inferior ST-elevation myocardial infarction (STEMI)
- ST-segment elevation >0.2 mV in two contiguous leads on electrocardiogram
- For inferior STEMI: and additional ST-segment depression in two contiguous anterior leads on electrocardiogram for a total ST-segment deviation (i.e. inferior ST-segment elevation plus anterior ST-segment depression) of ≥0.8 mV
- Persistent ST-segment elevation at the catheterization laboratorium
- <6 hours of symptom duration

**Exclusion Criteria**

- Cardiac arrest
- Previous acute myocardial infarction
- Previous coronary intervention (i.e. percutaneous cardiac intervention or coronary bypass grafting)
- Congestive heart failure
- End-stage kidney disease or hepatic failure
- Recent stroke
- Coagulopathy
- Pregnancy
- Killip class II – IV at presentation

**Reference**

Erlinge D, Götberg M, Lang I, Holzer M, Noc M, Clemmensen P, et al. Rapid endovascular catheter core cooling combined with cold saline as an adjunct to percutaneous coronary intervention for the treatment of acute myocardial infarction: The CHILL-MI trial: A randomized controlled study of the use of central venous catheter core cooling combined with cold saline as an adjunct to percutaneous coronary intervention for the treatment of acute myocardial infarction. 2014 May 13;63(18):1857–65.

## Healthy Controls

**Inclusion Criteria**

- Age >18 years

**Exclusion Criteria**

- History of cardiovascular disease, diabetes, or systemic disease
- Hypertension (defined as blood pressure of >140/90 mmHg)
- Cardiovascular medication
- Pathological electrocardiogram or CMR

**Reference**

Asgeirsson D, Hedström E, Jögi J, Pahlm U, Steding-Ehrenborg K, Engblom H, et al. Longitudinal shortening remains the principal component of left ventricular pumping in patients with chronic myocardial infarction even when the absolute atrioventricular plane displacement is decreased. 2017;17(1):1–9.
