# Supplementary material for: Association between left ventricular longitudinal function and left atrial strain in left ventricular dysfunction
Source: ESC Heart Fail. 2026 Feb 10;13(2):xvag046. doi: 10.1093/eschf/xvag046 (PMC12988773; doi:10.1093/eschf/xvag046)
Supplement: xvag046_Supplementary_Data [file xvag046_supplementary_data.zip › Supplementary Tables and Figures.docx]

**Supplementary Figures and Tables**

**Supplementary Table S1.** Medication in Study Participants

|  | HFrEF  (n=202) | CRT  (n=63) | IHD  (n=77) | All LV dysfunction  (n=342) |
| --- | --- | --- | --- | --- |
| Medication |  |  |  |  |
| - BB | 171 (85) ^a^ | 56 (89) | n/a | 227 (66) ^b^ |
| - ACEi/ARB/ARNI | 162 (80) ^a^ | 63 (100) | n/a | 225 (66) ^b^ |
| - Diuretics | 112 (55) ^a^ | 54 (86) | n/a | 166 (49) ^b^ |

Categorical variables expressed as n (valid %).

ACEi: angiotensin receptor inhibitors; ARB: angiotensin receptor blockers; ARNI: angiotensin receptor-neprylisin inhibitors; BB: beta-adrenergic blockers; CRT: candidates for cardiac resynchronization therapy; HFrEF: heart failure with reduced ejection fraction; IHD: ischemic heart disease; LV: left ventricular. n/a: not available. a: n=181, b: n=244.


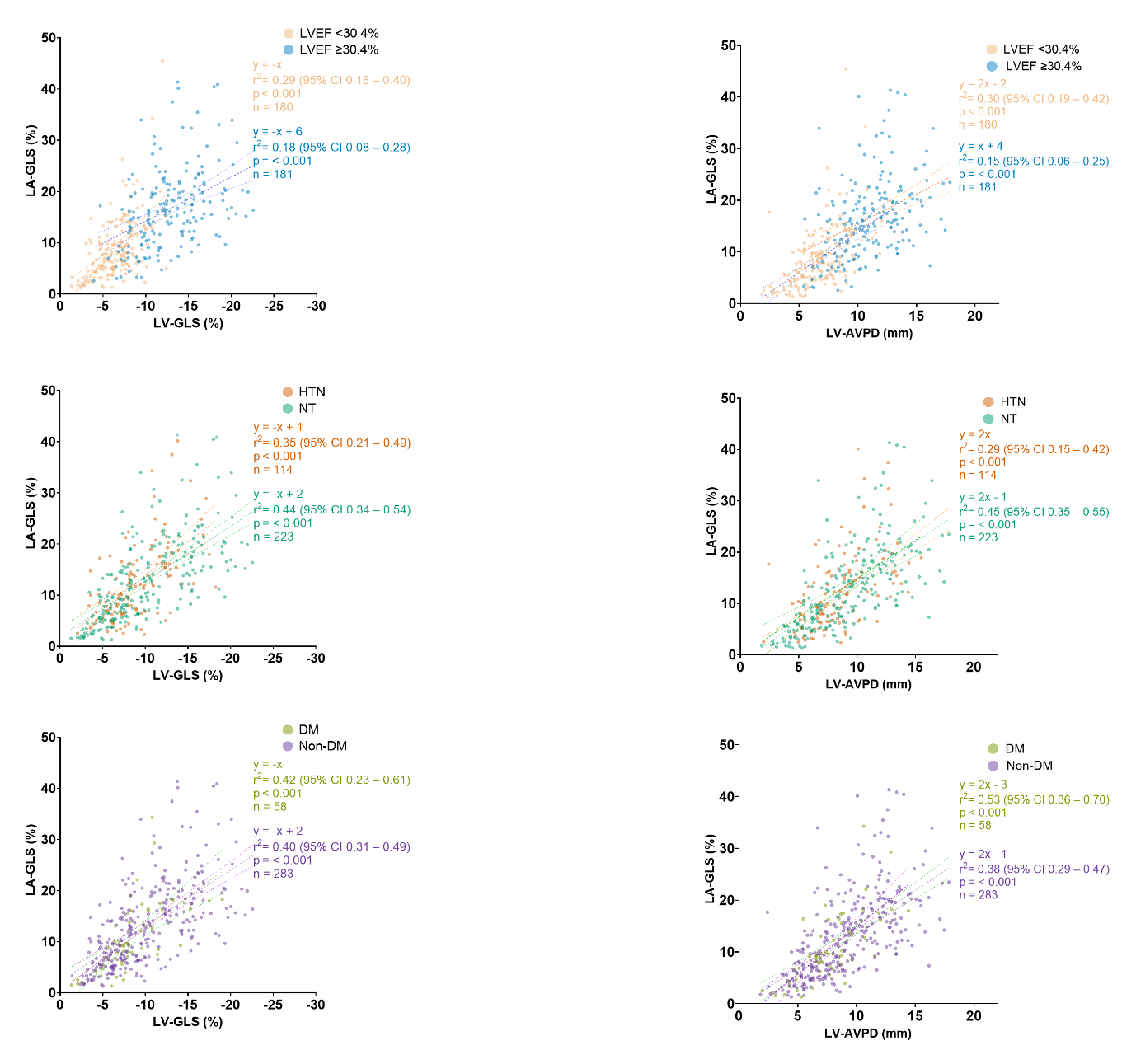


**Supplementary Figure S1**. Subgroup analysis of relationship between left ventricular and atrial function. Correlation between left ventricular (LV) global longitudinal strain (GLS) and left atrial (LA) GLS in the left column, and between LV atrioventricular plane displacement (AVPD) and LA-GLS in the right column. Top row: study participants with left ventricular (LV) ejection fraction (EF) below median vs study participants with LVEF at or above median. Middle row: hypertension (HTN) vs normotension (NT). Bottom row: diabetes mellitus (DM) vs non-DM. The regression lines with 95% confidence bands are indicated with dashed lines.

**Supplementary Table S2.** Intra- and interobserver variability in ten subjects.

|  | Intraobserver variability | |  | Interobserver variability | |
| --- | --- | --- | --- | --- | --- |
|  | Bias ± 1.96 SD (%) | ICC (95% CI) |  | Bias ± 1.96 SD (%) | ICC (95% CI) |
| LV-GLS (%) | 0.24 ± 1.27 (-1.00 ± 9.82) | 0.99 (0.97 – 1.00) |  | 0.71 ± 3.00 (-7.18 ± 32.14) | 1.00 (0.98 – 1.00) |
| LA-GLS (%) | -1.16 ± 4.90 (-3.56 ± 29.11) | 0.98 (0.93 – 1.00) |  | -2.29 ± 5.12 (-13.76 ± 25.52) | 0.96 (0.74 – 0.99) |

Intra- and interobserver variability expressed as absolute bias with relative bias (%) in parenthesis. CI: confidence interval; ICC: intraclass correlation coefficient; LA-GLS: left atrial global longitudinal strain; LV-GLS: left ventricular global longitudinal strain; SD: standard deviation.


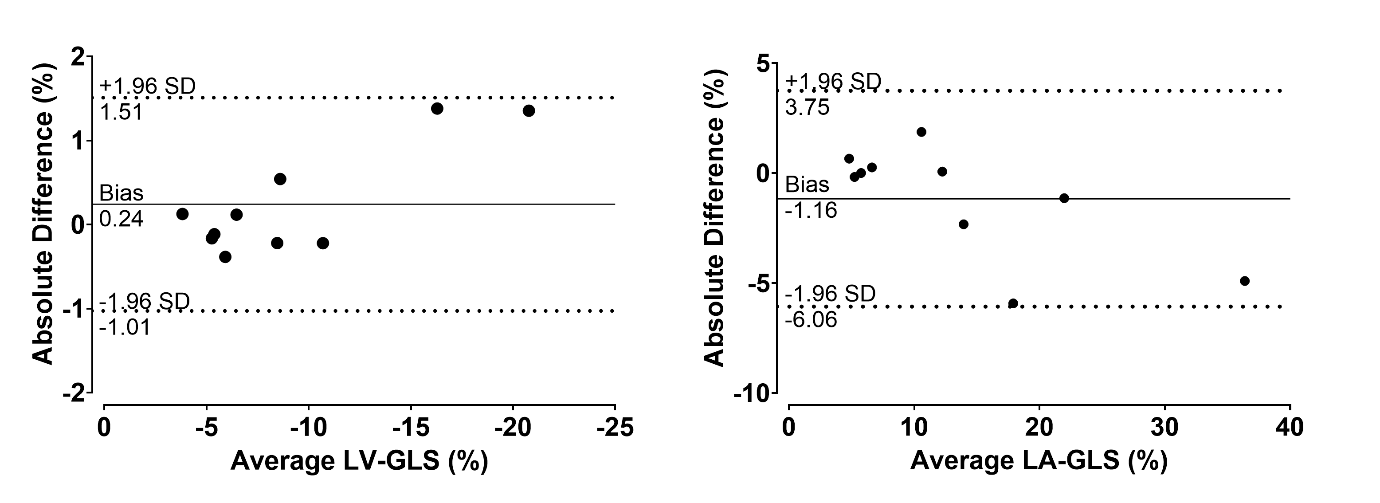


**Supplementary Figure S2**. Bland-Altman plots of intraobserver variability. Differences between observations of left ventricular (LV) global longitudinal strain (GLS) and left atrial (LA) GLS are plotted against the average. Observations were made by the same observer and >1 year apart. Means of the differences between measurements (bias) and limits of agreement (± 1.96 SD) are marked.


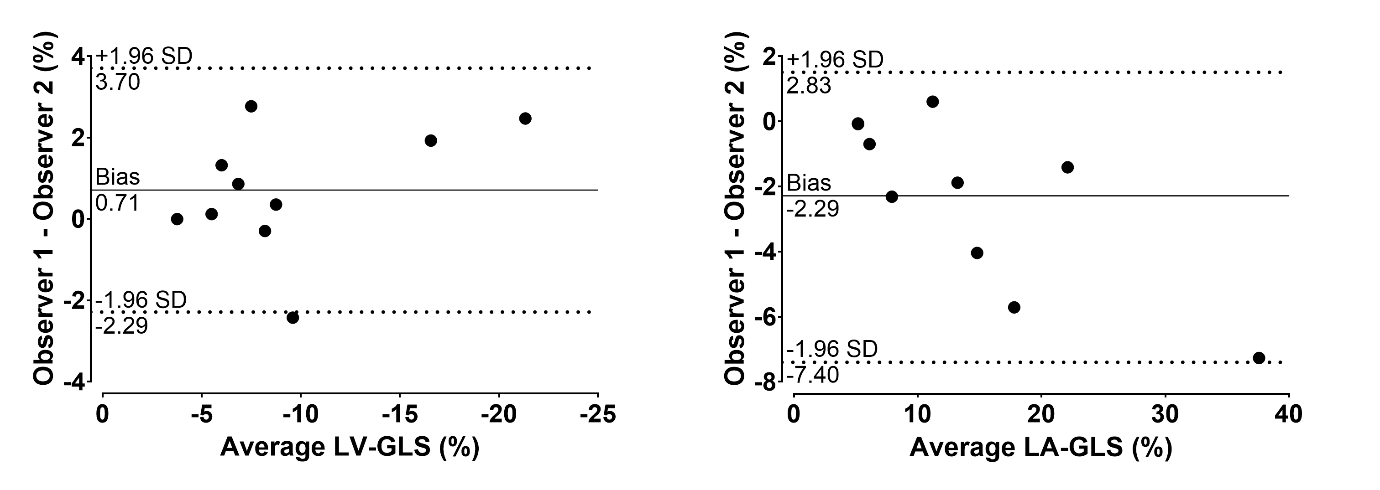


**Supplementary Figure S3**. Bland-Altman plots of interobserver variability. Differences between measurements of left ventricular (LV) global longitudinal strain (GLS) and left atrial (LA) GLS from two observers are plotted against the average. Means of the differences between measurements (bias) and limits of agreement (± 1.96 SD) are marked.
